# “There’s room to do more”: a mixed-methods study of the Temporary Assistance for Needy Families (TANF) diversion program and intimate partner violence in Georgia

**DOI:** 10.3389/fpubh.2024.1326467

**Published:** 2024-04-25

**Authors:** Tasfia Jahangir, Conjay Dahn, Ria Devakottai, Melvin D. Livingston, Briana Woods-Jaeger

**Affiliations:** Department of Behavioral, Social and Health Education Sciences, Rollins School of Public Health, Emory University, Atlanta, GA, United States

**Keywords:** intimate partner violence, welfare, policy, time-series, mixed-methods

## Abstract

**Introduction:**

Intimate partner violence (IPV) is a risk factor for homicides and suicides. As poverty is both a predictor and a consequence of IPV, interventions that alleviate poverty-related stressors could mitigate IPV-related harms. Temporary Assistance for Needy Families (TANF), a monthly cash assistance program, is one such potential intervention. In the state of Georgia, the TANF diversion program, which provides a non-recurrent lump-sum payment to deter individuals from monthly TANF benefits, is an understudied component of TANF that may influence the effectiveness of state TANF programs in supporting IPV survivors.

**Aim:**

This study quantifies and qualifies the role of Georgia’s TANF diversion program in shaping IPV-related mortality.

**Methods:**

This study relies on a mixed-methods sequential explanatory design. Using data from the Georgia Violent Death Reporting System (GA-VDRS), an interrupted time series analysis was conducted to estimate the effect of TANF diversion on IPV-related homicides and suicides. Semi-structured interviews were then administered with TANF policy experts and advocates, welfare caseworkers, and benefit recipients (*n* = 20) to contextualize the quantitative findings.

**Results:**

The interrupted time series analysis revealed three fewer IPV-related deaths per month after implementing TANF diversion, compared to pre-diversion forecasts (coefficient = −3.003, 95%CI [−5.474, −0.532]). However, the qualitative interviews illustrated three themes regarding TANF diversion: (1) it is a “band-aid” solution to the access barriers associated with TANF, (2) it provides short-term relief to recipients making hard choices, and (3) its limitations reveal avenues for policy change.

**Discussion:**

While diversion has the potential to reduce deaths from IPV, it may be an insufficient means of mitigating the poverty-related contributors to IPV harms. Its limitations unveil the need for improved programs to better support IPV survivors.

## 1 Introduction

Intimate partner violence (IPV), defined as “physical, psychological, or sexual abuse or aggression that occurs in a current or former romantic relationship” (1), is a pressing public health and policy concern. In its most severe forms, IPV can culminate in homicides or suicides of the victim, perpetrator or other individuals (i.e., corollary victims) (2). Since gender-based violence was decreed a political issue in the 1960s and 1970s (3), much of the public and legislative dialogue around government protections against IPV in the U.S. emphasized measures that were more reactive than preventive in nature. The most well-known of these include the Violence Against Women Act of 1994, which supported the criminalization of IPV and sought to equip victims with resources; the #MeToo movement, which increased awareness of sexual violence victimization; and, most recently, the ongoing advocacy for strengthening state-level anti-sexual assault statutes in response to Dobbs v. Jackson (2022), where the Supreme Court overturned the constitutional right to abortion (3–6). Relatively less attention has been paid to the factors that can *initiate* IPV, such as material hardship or economic stress (7–15).

A nascent body of both peer-reviewed and gray literature demonstrates how economic policies (such as the Earned Income Tax Credit, Section 8 housing vouchers, paid family leave, pandemic stimulus payments, and cash assistance from the Temporary Assistance for Needy Families program) can serve as primary and secondary prevention tools against various forms of violence (11, 16–26). Such efforts are critical for intervening early and curtailing violence before it begins or interrupting a cycle of violence. Additionally, because such policies are already in place in many cases, it can be resourceful and cost-effective to understand whether they have incidental effects on IPV (25) and elucidate possible areas for improvement to better respond to the needs of those in vulnerable circumstances. For instance, there is inconclusive evidence on whether TANF is currently reaching its full potential in addressing the needs of violence survivors (23). Examining the specific components of TANF may therefore allow researchers and policymakers to delineate the factors that promote or hinder TANF’s potential to support families and protect against IPV. This paper examines one such understudied policy in the state of Georgia: TANF diversion. As detailed in the literature review below, Georgia holds contextual value and public health significance for the study of welfare and IPV, given its notable prevalence of material hardship and violence victimization. Accordingly, this study aims to understand the role of TANF diversion in shaping IPV outcomes by (1) quantitatively estimating the effect of Georgia’s TANF diversion policy on IPV-related mortality with an interrupted time-series design and (2) qualitatively contextualizing Aim 1 findings through semi-structured interviews with key informants with TANF experience and expertise.

### 1.1 Literature review

The toll of IPV is both physical and psychological (1), impacting an estimated 10 million people in the U.S annually (27). In addition to being a significant public health problem that increases the risk of chronic disease, sexually transmitted infections, mental illness, substance use, and injury (28, 29), IPV is a risk factor for both homicides and suicides (2, 30). Roughly 1 in 5 homicide victims are killed by an intimate partner (1). Although studies on IPV-related suicides have largely taken place at state- and municipal-levels (31, 32), it is estimated that there may be over 2,900 IPV-related suicides occurring annually at the national level (31). Since IPV is underreported, even these grave prevalence figures likely underestimate the severity of the public health issue (33, 34).

**Figure 1:**
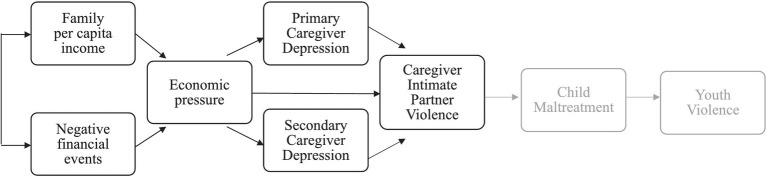
Family stress model adapted for violence (26).

Two decades of research demonstrate that poverty is both a predictor and a consequence of IPV, exerting mutually reinforcing effects (7–15, 35). For example, lower incomes may increase the likelihood of IPV exposure and IPV exposure may lower the survivor’s likelihood of remaining financially independent or escaping poverty (36, 37). This potential feedback loop suggests that interventions that alleviate poverty-related stressors could also be avenues for mitigating IPV-related harms. Indeed, 50 to 60 percent of IPV survivors participate in economic security programs (38), lending opportunities for intervention in such contexts. The Family Stress Model is a widely applied theoretical framework that can elucidate such levers for intervention; this model describes how financial stressors contribute to family economic pressure, which can impair mental health, and, in turn, produce relationship conflict or distress (12). The FSM has been directly applied to intimate partner violence (IPV) in a handful of studies (23, 24, 39), and an abundance of prior research implicitly demonstrates its applicability to IPV. For instance, there is evidence that economic hardship in the family can be a risk factor for caregiver depression, relationship dissatisfaction, relationship conflict, and aggression toward an intimate partner (12). Although the FSM extends beyond relationship conflict or IPV to issues related to child development, the present study focuses solely on IPV to better understand potential interventions for this specific pathway (Figure 1).

Temporary Assistance for Needy Families (TANF), a federal block grant program that provides monthly cash assistance to families in poverty, is one such intervention with the potential to reduce IPV-related harms (23, 40). However, the effectiveness of TANF has been subject to debate. In 1996, the United States Congress held a bipartisan agreement that welfare should neither disincentivize work nor promote dependency (41). This resulted in the passage of the Personal Responsibility and Work Opportunity Reconciliation Act (PRWORA), which concluded a 60-year-old program for qualified families to receive cash assistance (42). Legal researchers acknowledge that PRWORA dramatically reshaped the culture of public benefits in the United States, aligning with then-president Bill Clinton’s campaign pledge to “end welfare as we know it.” (41, 42). Specifically, one of the decreed objectives of the policy was to “end the dependence of needy parents on government benefits by promoting job preparation, work, and marriage.” (43) To meet this statutory goal, the New Deal-era cash assistance program, Aid to Family Dependent Children (AFDC) as well as other welfare programs were abolished, and TANF was introduced in their stead as a “workfare” program. TANF is a fixed block grant from the federal government that provides approximately $16.5 billion to states, the District of Columbia, U.S. territories, and federally recognized tribes. The stated goals of TANF are four-fold: (1) *Provide assistance to needy families so that children can be cared for in their own homes or in the homes of relatives;* (2) *End the dependence of needy parents on government benefits by promoting job preparation, work, and marriage;* (3) *Prevent and reduce the incidence of out-of-wedlock pregnancies; and* (4) *Encourage the formation and maintenance of two-parent families* (43). The block grant funding structure of TANF substantially differs from that of the AFDC, where the federal government contributed at least $1 in matching funds for every dollar states spent (43). In contrast, the TANF block grant transformed welfare into a program that afforded states considerable discretion on how they used their TANF funds (43). Furthermore, while the AFDC was almost exclusively a cash assistance program, states are free to use TANF funds for services and non-cash benefits (44). For example, besides cash, states can provide childcare vouchers and job training programs to those who qualify based on income and asset limits, as well as legal residency status (43). The discretion granted to states has led to wide variations in the use of TANF funds on basic cash assistance and reduced spending on basic cash assistance over time (43). For instance, state-level differences lie in who qualifies for TANF receipt, how much in cash assistance one can receive on a monthly basis, who is mandated to fulfill work requirements, if recipients are privy to benefit reductions for not fulfilling work requirements (i.e., sanctions), the maximum number of months recipients are eligible for benefits (i.e., lifetime limits), reductions in benefits after receiving payments for a certain period (i.e., benefit reduction limits), and penalties for having an additional child while receiving TANF (i.e., family caps) (23, 45, 46). This warrants additional research on specific components of TANF that may be helping or harming TANF’s potential to support families in general, and survivors of IPV in particular.

Diversion, a non-recurrent lump-sum payment aimed at diverting individuals from ongoing TANF benefits, is another component of TANF policy (45). In states such as Georgia, a diversion payment renders a TANF recipient ineligible for monthly assistance for up to 12 months; in others, the ineligibility period depends on the number of months’ worth of benefits the family received as a diversion payment (45, 47). The potential impact of TANF diversion on IPV is inconclusive because diversion has received less research attention compared to other TANF policy components, such as sanctions (23, 24, 48–51), and time limits (23, 24, 50, 52, 53). Currently, the District of Columbia and 32 states have a diversion policy in place, including Georgia (45). In 2020, a total of 642 individuals in Georgia received some form of diversion payment, and the average diversion payment per client was $168.72 (54), but can be as high as 4 months’ worth of cash benefits received through the regular TANF program (55). In contrast, the regular monthly TANF cash assistance payment is $223 for a family of three (or $2,676 per year if uninterrupted) (56).

Economic hardship and IPV are both pressing public health concerns in the state of Georgia. Its 14% poverty rate and $34,516 *per capita* income (55), coupled with its sharp 49% increase in IPV-related fatalities since 2020 (57) warrant policy-relevant solutions. The state experiences numerous racial and ethnic disparities in both poverty and IPV. For instance, the poverty rate of Hispanic, Black, and American Indian/Alaska Native individuals in Georgia are 19.7%, 20.3%, and 27% respectively, compared to the 9.5% poverty rate among White individuals (58). Additionally, Black women are disproportionately impacted by IPV in Georgia, at a rate that is 35% higher than that of White women and 2.5 times the rate of women of other races (59). As such, the state of Georgia deserves greater attention in the TANF literature to address these disparities.

Among the small handful of studies that do examine diversion policies, all but one (23) predate the last decade (60–64). Moreover, only one of these studies addresses Georgia, albeit limitedly, and the diversion policy discussed is different from the state’s present-day diversion program (64). Furthermore, only Spencer et al. (23) estimate the impact of diversion on IPV outcomes in 20 cities (with null results), but these are outside of Georgia. This study contributes to the literature by using evidence from Georgia to study the downstream influence of the current TANF diversion program on IPV.

### 1.2 Study hypothesis

Cash assistance programs and policies are widely held as effective anti-poverty measures that provide social protection and promote well-being (65). They can be lump-sum or recurring, and conditional versus unconditional (21). They operate in many countries across the world, with replicable evidence pointing to their capacity to inhibit IPV, even when such reductions are not an explicit objective of their programming (65, 66). For example, a review of 22 studies found that cash transfer programs in low- and middle-income countries (LMICs), whose focus was primarily poverty reduction, led to a decrease in some form of IPV (emotional, physical, sexual) in 73% of the cases examined (67). Similarly, a meta-analysis of 14 evaluation studies of cash transfer programs in LMICs found, on average, decreases in all types of IPV (68). In the context of contemporary American social policy, the effects of cash or near-cash transfers on IPV are less conclusive. While some studies have found that the Earned Income Tax Credit can improve the material well-being and relationship quality in low-income families (69, 70), others have not observed a relationship between EITC and IPV (16, 71). Relatedly, while studies in the early 2000s suggest that more generous TANF policies may be protective against IPV (72–75), a more recent analysis found that fewer TANF restrictions increased coercive victimization (23).

Similarly, in the present study, TANF diversion has the potential to either act as a facilitator or a barrier in reducing IPV-related harms. On one hand, a diversion payment can support IPV survivors with an immediate crisis without requiring them to undergo a strict, time-intensive application process to qualify for monthly TANF benefits. On the other hand, the reduced access to regular cash benefits may increase their financial strain and exacerbate IPV-related harms. As much of the evidence and the Family Stress Model (12) point to financial support as a protective factor for IPV, it was hypothesized that TANF diversion, which is aimed at reducing access to monthly TANF benefits, will increase the incidence of IPV-related deaths in Georgia.

## 2 Methods

This study utilized a mixed-methods explanatory sequential design (76) comprised of two phases: (1) an interrupted time-series analysis to estimate the effect of Georgia’s TANF policy on IPV-related mortality, and (2) semi-structured qualitative interviews with 20 key informants to contextualize the quantitative findings.

### 2.1 Phase 1 (quantitative): Interrupted time series design

#### 2.1.1 Data sources

The exposure of interest was the implementation of TANF diversion policy. The Urban Institute’s Welfare Rules Database (77) was referenced to determine July 2011 to December 2019 as the time period for analysis. Georgia’s ongoing diversion policy period began in February 2015. Before this, the state had another diversion policy in place from April 2006 to June 2011. Thus, July 2011 was used as the starting point to allow for a true “no policy” baseline, and December 2019 was used as an endpoint to avoid contamination of effects related to the COVID-19 pandemic. The dataset was split into two ‘before’ and ‘after’ periods based on the February 2015 start date of Georgia’s ongoing diversion policy. There were 1,278 observations in the 43 months prior to the implementation of the diversion policy (hereafter referred to as pre-diversion), and 1,579 observations in the 59 months following policy implementation (hereafter referred to as post-diversion).

The outcome of interest was intimate partner violence (IPV)- and intimate partner problem (IPP)-related mortality in the state of Georgia. Restricted state-level data on IPV- and IPP-related deaths, as well as decedents’ demographic information (age, sex, race, ethnicity), were obtained from the Georgia Violent Death Reporting System (GA-VDRS) through the Georgia Department of Public Health (78). The GVDRS consolidates data on violent deaths abstracted from death certificates, law enforcement records, coroners’ and medical examiners’ records, and Emergency Medical Services (EMS) reports. In this dataset, data are organized at the decedent level (i.e., one victim per observation). IPV- and IPP-related deaths were defined as homicides or suicides related to immediate or ongoing conflict or violence between current or former intimate partners. IPV- and IPP-related deaths were inclusive of corollary victims (for example, ex-husband kills his ex-wife’s new boyfriend, the child of an intimate partner, friend of the victim, or bystander). GA-VDRS defined an intimate partner as a current or former girlfriend/boyfriend, dating partner, ongoing sexual partner, or spouse, and is inclusive of same-sex partners. From July 2011 to December 2019. the dataset consists of 2,857 reports of IPV- and IPP-related deaths.

#### 2.1.2 Analysis

To understand the demographic makeup of the dataset, univariate analysis of race, ethnicity, gender, and age variables was conducted. An interrupted time series design estimated with an ARIMA (Autoregressive Integrated Moving Average) model was then used to analyze the effect of Georgia’s TANF diversion policy on reports of IPV- and IPP-related deaths. ARIMA is a modeling technique with a time-dependent outcome variable, a function of past counts of the variable and error values. It can be used for evaluating the impact of policy-level interventions on time-dependent outcomes as it controls for underlying trends, autocorrelation, and seasonality (79). It consists of four model components: autoregressive (AR) model, moving average (MA) model, seasonal model, and differencing. An ARIMA model is constructed by combining the four model components and is notated as ARIMA (*p, d, q; P, D, Q*). Here, p is the lag value of the AR component, d is the differencing interval, and q is the lag value of the MA component, and *P* is the seasonal lag value of the AR component, *D* is the seasonal differencing interval, and *Q* is the seasonal lag value of the MA component (79).

The model was used to examine the number of IPV- and IPP-related violent deaths at monthly time points from July 2011 to December 2019. Indicator variables for diversion were assigned to separate pre- and post-diversion data. The Box-Jenkins approach was followed (79), and an initial ARIMA model was developed to fit only the pre-diversion data. After establishing that the series was stationary prior to the introduction of TANF diversion, the optimal (*p, d, q; P, D, Q*) values for the ARIMA model were determined by examining the autocorrelation (ACF) and partial autocorrelation functions (PACF). Upon performing diagnostic checks of the residual ACF and PACF, the optimal (*p, d, q; P, D, Q*) values of the best-fitting model that achieved white noise were (0,0,3; 0,0,1)_9_. The ARIMA model was re-estimated for the entire time series, including the post-diversion data. A coefficient test was performed to estimate the effect of the diversion policy on the number of IPV- and IPP-related deaths.

### 2.2 Phase 2 (qualitative): Semi-structured interviews and thematic analysis

#### 2.2.1 Recruitment and consent

To contextualize the findings from Phase 1, in-depth semi-structured interviews (80) were conducted with key informants possessing experience and expertise in TANF. Eligible interviewees met one or more of the following criteria: (1) having a history of in-depth engagement with Georgia’s TANF policy through research and direct action, (2) bearing a professional responsibility to identify and refer eligible clients to TANF, or (3) being a current or former recipient of any TANF benefit in Georgia. Due to the recruitment challenges associated with a stigmatized, hard-to-reach group, as well as the rapidly declining population of TANF recipients in the state, eligibility criteria were not limited to TANF recipients with a history of receiving TANF diversion or experiencing IPV.

The study team’s existing relationships with community-based organizations and policy research institutes were instrumental in facilitating recruitment. Using purposive and snowball sampling methods (81), key informants were contacted from four child and family advocacy groups, one policy research organization, a school district, and a safety net hospital, all located in Georgia. Additionally, one interviewee was recruited from a policy research organization operating at the federal level. These initial touchpoints allowed the study team to engage TANF policy experts and caseworkers responsible for referring eligible individuals to TANF (e.g., *pro-bono* attorneys and a school-based specialist) as interviewees. The interviewees then disseminated a study flyer within their networks to aid the recruitment of current and former TANF recipients. TANF recipients contacted the study team via phone or email to express their interest and eligibility in participating in an interview. The final sample of interviewees consisted of six policy experts, three caseworkers, and 11 TANF recipients (*n* = 20).

All interviewees provided informed consent. Two members of the study team read a verbal consent document, provided an opportunity for interviewees to ask questions, and asked the interviewees to reiterate key components of the consent document to confirm their understanding of the study terms: *Would you describe in your own words what you are being asked to do? What would happen if you decided to stop the study?* Interviewees’ consent to participate and permission to record interviews were then documented. Following the interview, all interviewees received a $50 gift card as remuneration.

#### 2.2.2 Study instruments and data collection

All interviews were held over Zoom. Interviewees who did not have access to a computer joined the call using a teleconferencing number. To document the interviews, study team relied on Zoom’s record feature (preserving only the audio recordings) and professional transcription services. All interviews were anonymized. Table 1 summarizes the content of each study instrument administered during the interviews.

**Table 1:** Summary of study instruments administered during the qualitative interviews (Phase 2).

Instrument	Content	Policy experts	Case-workers	TANF recipients
Survey of sample characteristics	Age	**✓**	**✓**	**✓**
	Race and ethnicity	**✓**	**✓**	**✓**
	Education level	**✓**	**✓**	**✓**
	Years in current role	**✓**	**✓**	
	Marital status			**✓**
	Household size			**✓**
	Work history for the past two weeks			**✓**
	Other forms of government assistance			**✓**
	Perceived sufficiency of funds at the end of the month			**✓**
	Self-rated physical and mental health (1–10 scale)			**✓**
Interview guides	Personal/professional relationship to TANF	**✓**	**✓**	**✓**
	Knowledge/understanding of TANF diversion policy	**✓**	**✓**	**✓**
	Perceived benefits and harms of the TANF diversion policy	**✓**	**✓**	**✓**
	How diversion may impact survivors of IPV	**✓**	**✓**	**✓**
	Potential avenues for policy improvements	**✓**	**✓**	**✓**
	Slideshow and reflection of findings from Phase 1	**✓**		
	If and how clients are referred to diversion		**✓**	
	Relationship to finances			**✓**
	Reasons for applying for TANF			**✓**
	Learning about TANF			**✓**
	Overall impressions of TANF			**✓**
	Reflections on the utility of the diversion program (framed retrospectively for recipients of diversion payment(s), and hypothetically for others)			**✓**

***Surveys of Sample Characteristics***. Each interview began with an interviewer-administered survey via Qualtrics. Survey questions were tailored based on the grouping of the interviewee as a policy expert, caseworker, or TANF recipient (Table 1). All interviewees were asked about their age, race, ethnicity, and education level. TANF policy experts and caseworkers were additionally queried about the number of years in current role. The questions for TANF recipients were also tailored to include questions about marital status, household size, work history for the past 2 weeks, other forms of government assistance, perceived sufficiency of funds at the end of the month, as well as self-rated physical and mental health (1–10 scale).

***Interview Guides***. The survey of sample characteristics was followed by an in-depth semi-structured interview. Based on theory (23, 24, 39, 82) and prior research on TANF and violence (23, 24), three interview guides were developed for each group of interviewees: policy expert, caseworker and TANF recipient (Table 1). All interviewees were asked about their personal or professional relationship to TANF, their understanding of TANF diversion policy, perceived benefits and harms of the TANF diversion policy, and recommendations. Policy experts were delivered a slideshow of findings from Phase 1 and asked to reflect on the implications of the results in relation to their own knowledge and experience of TANF. TANF caseworkers were asked if and how they referred participants to the TANF diversion program. TANF recipients were queried about their relationship to their finances (i.e., their current financial support system, whether finances are a source of stress, their income in relation to their expenses), their reasons for applying for TANF, how they learned about TANF, and their overall impressions of TANF. In addition, two distinct sets of questions related to the diversion program were drafted for recipients, which were to be used based on their experience with diversion. For those who had received a diversion payment, a set of retrospective questions were developed to understand their experience and perceptions of the diversion program. For TANF recipients without exposure to TANF diversion, a set of hypothetical questions asked to reflect on circumstances where they would benefit from a one-time diversion payment over the monthly TANF schedule, and vice-versa. Since none of the recruited interviewees received a diversion payment, only the hypothetical questions were utilized.

#### 2.2.3 Analysis

Univariate analysis was conducted to summarize the data from demographic surveys. Interview transcripts were analyzed using an iterative thematic approach (83) in a series of steps. First, codes and subcodes were developed using a combination of inductive and deductive approaches. Inductive codes were borne out of the first four transcripts, whereas deductive codes stemmed from the interview guide. To ensure that the codes were meaningful and consistent, the first, second, and third authors collaborated on a codebook that standardized each code with definitions and constructs. The first and second authors then referenced the codebook to designate codes to all interview transcripts. To capture new concepts as they emerged, codes were revised iteratively until saturation (i.e., until the codes fully represented all the relevant information in the transcripts). Two coders then coded each transcript and met to reconcile codes and resolve discrepancies. Based on the patterning of the codes, salient themes were derived and substantiated with quotations.

## 3 Results

### 3.1 Quantitative Phase 1: Interrupted time series design

#### 3.1.1 GA-VDRS sample description

Table 2 summarizes the demographic makeup of Georgia’s IPV and IPP mortality data reported on the NVDRS from 2011 to 2019.

**Table 2:** Demographic characteristics of IPV and IPP-related mortality cases in the GA-VDRS.

Variable	Value
**Age, mean (SD)**	40.46 (14.54)
**Gender** **^1^** **, *n* (%)**	
Male	1999 (69.67)
Female	858 (30.03)
**Race, *n* (%)**	
White	1948 (68.18)
Black or African American	760 (26.60)
Asian	5 (0.18)
Native Hawaiian or Other Pacific Islander	56 (1.96)
American Indian or Alaska Native	57 (2.00)
Unspecified	31 (1.09)
**Ethnicity, *n* (%)**	
Hispanic or Latino	4.66 (131)
Unspecified	1 (0.04)

1 GVDRS labels as “sex at time of incident,” with options “male,” “female,” “unknown.”

#### 3.1.2 Findings from interrupted time series analysis

**Figure 2:**
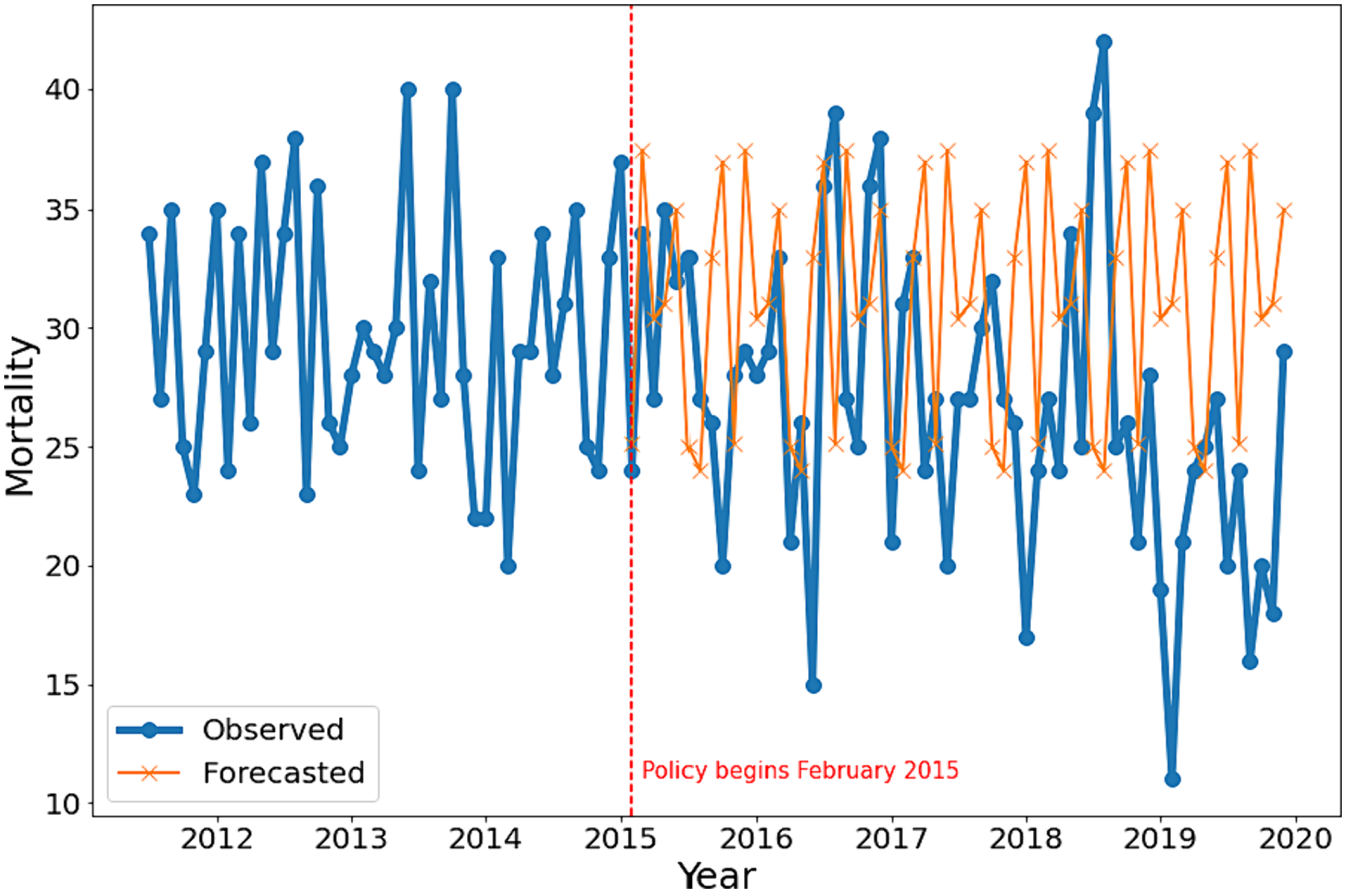
Comparison of observed and forecasted IPP and IPV-related mortality before and after TANF diversion policy implementation in 2015 (2012–2019).

With the inclusion of post-diversion mortality data, the ARIMA (0,0,3 0,0,1)_9_ model revealed 3 fewer observed deaths per month, compared to pre-diversion forecasts (coefficient = −3.003, 95%CI [−5.474, −0.532], *p* = 0.017). As such, the findings did not support the study’s initial hypothesis (i.e., that diversion will result in an increase in IPV-related mortality). Figure 2 illustrates the change in IPV- and IPP-related mortality trends after the 2015 diversion policy and compares the forecasted pre-diversion mortality trend to the observed post-diversion mortality trend.

### 3.2 Qualitative Phase 2: Semi-structured interviews

#### 3.2.1 Interviewee sample description

Table 3A summarizes the demographic information of the policy experts, caseworkers and TANF recipients interviewed about TANF diversion. Table 3B provides information on additional details gathered from TANF recipients.

**Table 3A:** Demographic information of all interviewees.

	Policy experts (*n* = 6)	Caseworkers (*n* = 3)	TANF recipients (*n* = 11)
**Number of years in current role, mean (SD)**	4.83 (4.61)	11 (12)	N/A
**Age, mean (SD)**	44.17 (11.44)	43 (19.08)	26.9 (5.54)
**Race/Ethnicity, n (%)**			
Asian American or Pacific Islander	0	1 (33%)	
Black or African American	3 (50%)	0	9 (81.82%)
White	2 (33%)	2 (66%)	1 (9.10%)
Mixed	0	0	1 (9.10%)
Other	1 (17%)	0	0
Hispanic or Latino	1 (17%)	0	0
**Education Level, n (%)**			
No formal schooling	0	0	1 (9.10%)
Some high school	0	0	1 (9.10%)
High school diploma	0	0	1 (9.10%)
Some college or 2-year degree	0	0	5 (45.45%)
Bachelor’s degree	0	0	2 (18.18%)
Graduate or professional degree	6 (100%)	3 (100%)	1 (9.10%)

**Table 3B:** Additional characteristics of TANF recipients.

Variable	Value
**Household size, mean (SD)**	3.27 (1.10)
**Worked for pay in the past 2 weeks, n (%)**	6 (54.45%)
**Applied or received other sources of public assistance in the past 12 months, n (%)**	
Supplemental Nutrition Assistance Program (SNAP)	9 (81.82%)
Supplemental Security Income (SSI)	6 (54.45%)
Social Security Disability Insurance	1 (9.10%)
**Finances at the end of the month, n (%)**	
Some money left over	2 (18.18%)
Just enough to make ends meet	1 (9.10%)
Not enough to make ends meet	7 (0.64%)
**Self-rated mental health (1–10 scale), mean (SD)**	6.55 (1.81)
**Self-rated physical health (1–10 scale), mean (SD)**	7.73 (1.49)

#### 3.2.2 Findings from thematic analysis of semi-structured interviews

##### Theme 1: Diversion as a “band aid” solution for the access barriers to receiving monthly TANF payments

###### Subtheme 1.1: Diversion disincentivizes seeking public assistance

Despite the quantitative findings on the protective effects of diversion payments on IPV-related mortality, diversion payments were largely considered unfavorable by interviewees because they offered a smaller one-time payment than what the recipients would have received with regular TANF payments over the course of a year.

Caseworkers described diversion as a deliberate effort to turn individuals away from receiving their fair share of public assistance:

*“Cynically, it is an effort to pay off poor people with one little bit of money, foregoing some other little bit more money.”* (Caseworker)

Multiple interviewees described that there was little benefit to receiving a small amount of assistance through a one-time TANF diversion payment:

*“For my clients to benefit from TANF, the amounts need to be livable [….]. My clients need easier accessibility. My clients need childcare. My clients need child support services. My clients need accessible healthcare and resources that help them with their food insecurity. Diverting them to try and put a lump sum of some smaller amount [….] would not be helpful for my clients. [….] I can’t see any helpfulness except from my clients’ perspective that any funds to help them immediately is better than the anticipation of long-term help, which they never see.”* (Caseworker)

###### Subtheme 1.2: Potential harms of diversion

According to some policy experts, the diversion program may even be harmful because it disqualifies TANF recipients from accessing other TANF benefits and the monthly TANF payments for the next 12 months:

*“To have the one-year pause seems like it’s a lot. It seems like that might be overkill. If there was a way to lessen that, I think it might be beneficial. I mean, again, the reason why people are in the program is because they are needy. That’s the N part of [TANF]. To think that this one-time payment is going to overcome the year in the future? I do not know. I just think that that’s too long.”* (Policy Expert).

According to one interviewee, the harms of TANF diversion go beyond losing access to monthly cash payments:

*“What happens when you get a diversion payment is you lose access to some of the other services that TANF provides. So, if there is case management services, if there is childcare assistance, if there is help with the things you need to go to work, you lose access to all of those. So, what you’re getting is a short-term cash payment, but nothing else.”* (Policy Expert)

Another interviewee also perceived TANF diversion to have harmful implications from a broader population health perspective:

*“I could see it deterring health equity. I think whenever you have these programs that say, “I’m happy to help you now, but that means I can’t help you in the future”. People who are going to take you up on that offer are going to be the ones that are the most vulnerable. And, by definition, [….], they’re the highest risk for health disparities and health inequities. So I definitely feel like this has a potential to be harmful, just even despite seeing your graphs about the deaths.”* (Policy Expert)

###### Subtheme 1.3: Barriers to accessing traditional TANF payments

Interviewees also highlighted multiple barriers to receiving the “traditional” monthly TANF payments, suggesting that this was not an easily accessible resource. One policy expert described how such barriers may be particularly detrimental to IPV survivors:

*“Georgia is famous for having really extreme barriers in order to access cash. And it’s really unfortunate because if you are in a situation where you are potentially under threat of violence or have already experienced violence, […] moving quickly and accessing resources quickly to either get out of that circumstance is essential so that that’s the harm, basically, you have this resource, but you’re making it putting up so many barriers that it’s almost as if the resource may not available to you, right, if you don’t need these certain conditions. And to me, [that] should not be the point of a cash assistance program.”* (Policy Expert)

Logistical hurdles during the TANF application process, such as depending on public transportation to the Department of Child and Family Services (DCFS) and lengthy office wait times, made the application process itself more difficult to access. Recipients also noted the unfavorable conditions of the DCFS facilities, lack of resources, and unreliable staff assistance as additional barriers, citing employee burnout and insufficient staffing as potential reasons for the difficulties during the process:

*“I’m very serious when I say I think they are burned out and no one wants to do that job anymore because I remember standing in lines where women have two or three children. It’s hot, they have barely any AC. The lines are out the door. And then the computers break down.”* (TANF Recipient)

Application completion and processing times were cited as barriers to accessing the monthly TANF benefits. One participant recounted the length of time it took for them to complete the application, and the time it took for them to receive an update on their application status:

*“If people still are getting benefits, I would like to see how they’re doing it. Maybe they’re taking a whole day off to go there because that’s basically what you have to do now. You can’t just go in for 30 minutes and leave. It’s a whole day job going to the DFCS [Division of Family and Children’s Services] office. […] It took like a whole – like a month for them to process everything and then for them to send me out a letter to tell me when I was approved. It took like 30 days.”* (TANF Recipient)

Additionally, recipients described excessive documentation requirements, including those that may not be readily on-hand, which delayed their time to complete the application:

*“You’ve got to have, first, the kids’ information, like their birth certificates, social security numbers, stuff like that. I can’t say it was easy. […] When I was doing [the application] I did get a little frustrated, ’cause I was like, […] “dang, y’all ask for so much stuff. Why y’all ask for all this stuff?” And I had to take stuff back up because some stuff I didn’t have at the time; I had to go get it and take it back up there. So that really made the process even a little longer.”* (TANF Recipient)

Many recipients expressed being denied TANF benefits multiple times and having to complete two or more applications before being approved. Interviewees were dissatisfied by caseworker communication and the extensive amount of time it took to be followed-up with on their application. One interviewee also described never being contacted about their application status or the reason for the final decision:

*“Oh my God. It was kind of rough and stressful. Cause the first time that I applied a caseworker never called and contacted me. She didn’t ever get in contact. And I checked my gateway account and I was denied. But she didn’t ever tell me why. So it was stressful cause I could never get in contact with her.”* (TANF Recipient)

###### Subtheme 1.4: Diversion as an alternative (albeit imperfect) solution for barriers to accessing traditional TANF payments

Given these and other potential hurdles to receiving monthly TANF payments, policy experts contended the one-time diversion payment be a more readily-available alternative in these circumstances:

*“The hassle factor in TANF programs is really high and significant. And so, [diversion] gives families who need small amounts of income […] a better source of help than going through the onerous requirements that what they’d otherwise have to go through.”* (Policy Expert)

Interviewees deemed TANF diversion as a possible mechanism to overcome eligibility criteria that may not always be easy for IPV survivors to meet, such as work requirements:

*“So, I think [diversion] could be helpful for families who have pretty significant barriers who can’t meet the work rate. So, they’re going to lose assistance, then they might actually get some assistance rather than not getting anything.”* (Policy Expert)

Thus, although TANF diversion in and of itself is not a desirable policy, the challenges associated with receiving the monthly TANF payments suggest that TANF diversion might be operate as a “band aid” solution to these barriers. This may explain the findings on the protective effect of diversion on IPV-related mortality in Phase 1, as suggested by policy expert interviewees:

*“Georgia’s TANF program is so horrendous in terms of allowing people to access it […] because their program is so bad that diversion payments actually offer an alternative.”* (Policy Expert)

##### Theme 2: Diversion as short-term relief to recipients making hard choices

All interviewees agreed that the main benefit of diversion, especially to victims of IPV, is that the one-time payment may overcome some of the hurdles of the regular TANF application by providing quicker assistance. One caseworker explains the need for IPV victims to have immediate access to resources:

*“We have discovered that victims of domestic violence need the financial resources they can gather before they can leave. The fewer resources that they have at their fingertips, the less likely it is that they and their children will be able to escape beatings, abuse, and murder without those resources.”* (Caseworker)

Other policy experts described how diversion can play a role in providing this short-term relief:

*“If we’re thinking about people who are in crisis and need access to cash supports, diversion is one mechanism that could be helpful. So instead of going through, which might be perhaps more a little bit more rigorous of an application process, diversion could be a way to more quickly get access to those cash supports to help somebody in crisis to quickly just address needs of safety and economic stability.”* (Policy Expert)

Another interviewee similarly described the temporary utility of this relief in assisting an IPV survivor escaping crisis situations:

*“I imagine that our patients do need cash assistance, especially because a lot of times people need safety transfers. They come in and they’re injured close to their home or someplace where they don’t feel safe going back and they do need cash assistance to help them out of that situation and get rid of those environmental stressors. I can see where the benefit would be just to have this money easily or hand it to them. That’s the only benefit because I think in the long term, if they’re not having a whole year after that, it can be pretty detrimental, especially if people are relying on that assistance. I think the risk will outweigh the benefit, though, in the long term.”* (Policy Expert)

This was corroborated by a TANF recipient who suggested that diversion can help survivors transition away from dire circumstances:

*“Since they’ll try to use these funds to make their ends meet, at least they can settle with it and at least move on from their problems or what they have gone through.”* (TANF Recipient)

Beyond this, it was challenging for interviewees to perceive other, more long-term benefits to diversion. One caseworker suspected diversion to be a mechanism of absolving the TANF program of its responsibilities to provide for families in the long-term:

*“This sounds like a big cost-cutting effort that would prey on desperately needy and desperately poverty-stricken women who need money immediately to feed their children or get them through some kind of emergency. I would assume that was the purpose of it and to cut the cost of it.”* (Caseworker).

Consistent with the quote above, caseworkers contended that diversion particularly affects individuals making hard choices. During these periods of vulnerability, individuals may opt for quick access to the one-time payment, even if the dollar amount is lower than what they may have received over the course of 12 monthly payments:

*“Exactly. If you have to pay your rent, you have to do what you have to do to keep yourself and your child from going homeless. It’s not a hard choice. You would make it. I would make it. Any parent would make it to keep their child from being homeless or from being hungry or from being sick.”* (Caseworker).

These difficult circumstances were similarly acknowledged by another interviewee:

“*I think about the families that I serve, if you’re stuck between a rock and a hard place, you are likely going to take this big lump sum, I would think.”* (Caseworker)

###### Theme 3: Limitations to TANF diversion reveal avenues for policy change.

There was consensus among interviewees that Georgia’s TANF program, and diversion specifically, are fraught with limitations. Accounting for these challenges, TANF recipients, caseworkers, and policy experts shared several avenues for policy advocacy to improve the material conditions of IPV survivors. Some proposed ways of improving Georgia’s implementation of TANF, whereas others cited policy alternatives that may be better suited for curtailing IPV. Table 4 summarizes these recommendations.

**Table 4:** Avenues for policy advocacy identified by interviewees.

Category	Recommendation	Example quote(s)
**TANF-specific**	Increase the dollar amount of both one-time and recurring cash payments	*“I think for my client population, the amounts are so low monthly. If the amount could be increased...”* (Caseworker)
		*“TANF, being a block grant, it has not increased. Even social security gets a small cost of living adjustment and the amounts of TANF have not increased.”* (Caseworker)
		*“I mean, think certainly we can do more research, but I think there is indications that point to get access to cash, one has positive outcomes and we do not need to have so many barriers in place to getting the access to cash. And in fact, the barriers may undermine the improvements that the cash itself could have.”* (TANF Expert)
		*“I cannot foresee any negative consequences about giving people money, except that it just would not be enough.”* (TANF Expert)
		*“Just having a decent lump sum, like a thousand or a couple thousand dollars to get you over whatever this hump is, whether it be rental arrears or paying a credit card down. So your credit score will go up and then you can be eligible to buy a home. These are the things that actually generate wealth. These are the things that keep people safe. The number one reason that people return to abusive situations is based on economic precarity. And when people do not have to worry about how they are going to be housed, where their income is coming from, whether or not they are going to be able to take their care of their child, they are so much less likely to return to abusive situations. So yeah, if it were funded well, I think it could do a lot of good.”* (TANF Expert)
	Allow recipients of TANF diversion to continue accessing monthly TANF benefits	*“Having something that’s similar to this crisis payment, but not without the repercussions of that diversion payment that somewhere you are disqualified for TANF..for monthly benefits.”* (Caseworker)
	Improve case management by investing in manpower and resources	*“Just trying to really invest in the people who work there, invest in their employees, invest in […] the community. […] Now you cannot even talk to anyone on the phone. I do not know how people are still getting benefits.”* (TANF Recipient)
		*“Georgia needs to do better with resources. They need to do better with case management. When it comes to benefits they need to just to do better. They need to do better, they need to do better. And they need to start putting their clients’ best interests, instead of getting mad that the clients see the benefits”*
	Destigmatize TANF	*“If you think about what it takes to prove all of the different things that they are asking […] to prove that you are compliant with the work requirements or the other “acceptable” activities, the way that these DFCS workers are living in poverty themselves essentially, and are often the people receiving the brunt of the frustration about how the system is working, and how then that informs how they treat the people that they are interacting with. So, in addition to the policies, there’s the actual practice and that is another layer of how harmful the experience can be for people […] So, even people who do potentially qualify do not receive it or they think it’s not worth it, because it’s so painful […] And if we thought about it in terms of everybody needs a little bit of help sometimes and we should make it feel good and feel like a community, And so that’s the way that I envision like how we could talk about these benefits and make them feel good for people. The more that we can shift the rhetoric around it I think it will inform the policy.”* (Policy Expert)
		*“The stigmas and the other pieces that are heavily associated with cash have created the very weak program that we have today.”* (Policy Expert)
	Conduct more research to identify and tackle barriers	*“Because there are a lot of harmful policies in TANF that you can focus on hat I think it would be interesting to parse out. Like which of the policies are most harmful? Then that can inform policy change.”* (Policy Expert)
		*“I think that research done with near misses basically. Like people who almost qualified for TANF, but did not,* versus *people who have qualified for TANF. And seeing what the qualitative research around what their quality of life looks like because they did not receive it or because they did receive it would be helpful in for us in terms of being able to share with policy makers, “This is the difference TANF can make for a family.””* (Policy Expert)
		*“And I think that programs like TANF and a diversion payment and all of this, like they are great, but I think that more data and information needs to be out. […] I just think about all of these support programs and they are great, but there’s room to do more.”* (Caseworker)
**General**	Take a preventive, non-adversarial approach by accounting for the material and structural drivers of IPV	*“We were focusing pretty heavily on policies that kind of protect the physical bodies of survivors. And there was a very heavily criminal legal component to it. And we have been making the shift over the last maybe four or five years towards non carceral solutions and looking at the real drivers of domestic violence, which tend to be the more structural issues, more things like access to healthcare or lack of affordable housing, lack of a living wage, all of those things are the biggest drivers of gender-based violence.”* (Policy Expert)
	Raise the floor for wages	*“I do believe that this entire government should at least -- if they are not going to be dependable with these resources --then they should just increase the wages for people so that at least with mothers who are single, they are making enough money.”* (TANF Recipient)

## 4 Discussion

Findings did not support the hypothesis that diversion will increase the number of IPV-related deaths in Georgia. Instead, three fewer deaths per month were observed after the implementation of TANF diversion. However, the qualitative findings suggest that diversion (1) is a “band-aid” solution to the access barriers associated with TANF, (2) only provides short-term relief to recipients making hard choices, and (3) has limitations that reveal avenues for policy change.

Our quantitative findings suggest that TANF diversion in Georgia carries the potential to reduce IPV-related harms. These findings stand in contrast to the literature demonstrating the protective effects of ongoing cash assistance on IPV (11, 16–26). Further investigation is necessary to determine whether TANF diversion is only reducing the escalation to death in incidences of IPV, or mitigating IPV more broadly.

As documented previously, there are numerous hurdles to receiving TANF benefits in Georgia, including but not limited to stringent eligibility criteria (84), 45-day-long application processing times (85), 30-hour work requirements, and 48-month time limits (43). This is also evidenced in the historically low TANF-to-poverty ratio in Georgia, wherein for every 100 families living in poverty, only five receive assistance through TANF; this TANF-to-poverty ratio has declined 77 points since the mid-1990s (86, 87). Indeed, in 1994, there were 141,596 families in Georgia receiving TANF assistance; last year, in 2022, only 5,734 families received assistance – a 96% decline in TANF receipt (88). Therefore, for many, a diversion payment may be the only route for cash assistance, and it cannot be assumed that monthly TANF payments are a readily available alternative. Additionally, to receive a diversion payment, an individual would not have to subject themselves to the potentially challenging work requirements associated with the recurring monthly TANF benefits (89), which may facilitate access to cash benefits. These lower barriers to accessing diversion payments relative to traditional TANF monthly benefits may potentially explains some of the protective effects observed in the time-series analysis.

Coupled with the results from the time-series analysis, the qualitative findings on the role of diversion as short-term relief suggest that many individuals may opt for a diversion payment to curb an acute stressor before their challenges intensify, such as emergency assistance to pay rent, utility bills, repairs, other housing- or vehicle-related costs, or domestic violence services. This has been suggested by other examinations of TANF diversion at the national level (47). Additionally, there is broad recognition among psychologists that IPV is associated with psychological stress of varying intensities and durations (24, 90–93). For example, IPV survivors may endure long-term or chronic stress from continual violence and intimidation, as well as short-term stressors that culminate over time, such as becoming unemployed or lacking the transportation to escape (24, 93–95). Therefore, administering short-term interventions have been identified as an important element of coordinated community responses to IPV (96). However, the current evidence on short-term IPV interventions prioritizes psychotherapeutic modalities and shows greatest promise for intrapsychic needs – and even then, the effects of these short-term interventions are known to attenuate over time (96). While there is some exploratory evidence on the role of small amounts of cash for short-term (yet insufficient) relief among IPV survivors who are TANF recipients (24) and women living with HIV (97), there is a need for additional research to conclusively determine whether quick material support (such as a one-time payment) can specifically function as a short-term intervention against acute stressors. It is also critical to examine how these short-term resources can be paired with more durable, long-term interventions that relieve more chronic concerns and sustain the well-being of IPV survivors.

While some scholars have coined TANF a failure due to its limited reach and the barriers noted above (41, 86), TANF’s past and present suggest that the policy may be functioning as intended, with unlimited discretion at the state level. Considering (a) the program’s original goal of keeping families off welfare rolls (98) without accountability for ensuring their self-sufficiency (41), (b) its efforts to divert individuals from receiving monthly benefits (45), (c) its marginalization of Black and Latinx families (87, 99), (d) states’ redirection of TANF funds to other programs (100), (e) states’ accumulation of TANF surplus funds, (e.g., $2.2 million in Georgia) (87) and (f) the paucity of federal oversight as states carry out these activities (100, 101) suggests that TANF’s inertia in lifting families out of poverty may be systemic. Indeed, TANF closely represents neoliberal philosophy: government responsibility is relegated and decentralized to lower administrative units that determine the roles and implementation, and eligibility for aid is determined through the lens of economic productivity and exchange (i.e., work requirements), rather than broader social and systemic forces (102). Accordingly, TANF should not be considered a panacea for alleviating poverty (101). However, it is one of the only income-support programs of its kind in the U.S. since Unemployment Insurance and Supplemental Security Income have more precise eligibility criteria, and EITC would be insufficient as the sole anti-poverty program. Because TANF still provides relief to a small proportion of families in poverty, it is important not to abandon the policy without introducing structural reforms that remedy the inequities and material conditions forcing families to seek TANF in the first place.

### 4.1 Limitations and strengths

Although this study moves the TANF literature forward by examining an understudied policy component, several limitations must be acknowledged. Beyond the possibilities noted above, other unexplored factors may be shaping the relationship between TANF diversion and IPV-related mortality. With limited data availability, and the inability to randomize diversion payments, an interrupted time-series design was the most robust alternative for examining the outcomes of interest. The results from this analysis may be nullified should other confounders occur near the time that TANF diversion policy went into effect. Of note, the COVID-19 pandemic assistance relief is not one of these confounders, as the study does not use mortality data from 2020. One potential confounder may be state legislation that extended unemployment benefits to IPV survivors in 2015 (103). As more data becomes available, future research should model the effect of both policies simultaneously.

Additionally, the study only examines TANF diversion in Georgia. Because the association between TANF diversion and IPV-related mortality may vary by contextual factors and state-level differences in the implementation of TANF policy, findings may not be generalizable to other states implementing a TANF diversion policy. As such, future research should replicate these analyses in other states.

Despite these limitations, there are multiple strengths to this study. Population-level studies of the impact of social and economic policies on violence are only recently receiving research attention in the U.S. The study contributes to this growing body of evidence by investigating a specific element of a welfare policy that can influence its effectiveness in supporting disadvantaged families. Because TANF is a complex program, malleable to social and political conditions at the state level, this natural experiment lends an opportunity to evaluate the impact of a TANF policy component within the “real world.”

Additionally, the focus on mortality data in the interrupted time series analysis responds to a recent call in the injury and violence field to examine the forms of IPV that culminate in lethal outcomes (2). According to this call, these instances of IPV represent missed opportunities to intervene before the escalation to fatalities, either due to ineffective interventions or a complete lack thereof (2). These fatal cases of IPV, therefore, deserve greater research attention to identify alternative mechanisms of prevention. However, the field may also benefit from additional research that characterizes the effect of TANF diversion on incidences of IPV that do not necessarily result in deaths to clarify whether the program prevents IPV more broadly, or merely its escalation. The qualitative work for the present study can lay the groundwork for understanding potential mechanisms that may also apply to non-fatal forms of IPV.

Overall, this analysis is strengthened with the mixed-methods approach. Although quantitative methods such as ARIMA modeling can serve as robust tools for examining whether specific policies can impact health outcomes, they do not necessarily capture complex phenomena in their entirety. In these instances, combining quantitative and qualitative methods with a mixed-methods design can allow researchers to contextualize and explain quantitative findings.

## 5 Conclusion

This study estimated the role of Georgia’s TANF diversion policy in shaping IPV. There was an observed decrease in the IPV-related morality in Georgia after the TANF diversion policy went into effect. However, policy experts, caseworkers, and TANF recipients engaged in this study revealed that the TANF diversion policy is likely fraught with limitations, despite the short-term relief it may provide to vulnerable recipients. Few studies examine the impact of social and economic policies on violence-related inequities. This study underscores the importance of paying close attention to the caveats of social policy, wherein seemingly inconsequential or previously unobserved policy elements can have critical implications for the health and well-being of families in poverty. It also highlights the importance of context: no two state-level TANF policies are alike, and state-level case studies of TANF policy components are vital for proposing tailored interventions and policy alternatives.

While this study elucidates the potential implications of TANF diversion for violence prevention, it is merely a starting point. More information can be gleaned by comparing the effects of TANF diversion policy to that of other states and states that have no TANF diversion programs in place. Future work may also consider examining the effects of the program on non-fatal IPV, as well as other forms of violence (e.g., community violence). It may also be valuable for the field to understand whether TANF diversion has differential effects across demographic groups.

Lastly, the benefits of community-engaged research for triangulating qualitative and quantitative data are widely recognized (104, 105). Policy researchers are encouraged to tap into these strengths, while broadening their definition of “experts” to account for communities beyond the research setting that frequently interact with policies of interest. Such an approach may facilitate a stronger understanding of the social mechanisms observed in natural experiments.

## Data availability statement

The dataset from Phase 1 is not readily available because an application to the Georgia Department of Public Health is required to obtain the data. Deidentified qualitative data from Phase 2 can be made available upon reasonable request. Requests to access the datasets should be directed to tasfia.jahangir@emory.edu.

## Ethics statement

The study involving human participants were reviewed and approved by the Emory University Institutional Review Board. Participants of Phase 2 provided their verbal informed consent to take part in this study.

## Author contributions

TJ: Conceptualization, Formal analysis, Methodology, Project administration, Writing – original draft, Writing – review & editing. CD: Formal analysis, Writing – original draft, Writing – review & editing. RD: Formal analysis, Writing – review & editing. MDL: Conceptualization, Formal analysis, Resources, Supervision, Validation, Writing – review & editing. BWJ: Conceptualization, Resources, Supervision, Validation, Writing – review & editing.
